# Supplementary material for: Location, Age, and Antibodies Predict Avian Influenza Virus Shedding in Ring-Billed and Franklin’s Gulls in Minnesota
Source: Animals (Basel). 2024 Sep 26;14(19):2781. doi: 10.3390/ani14192781 (PMC11475586; doi:10.3390/ani14192781)
Supplement: Supplementary file 1 [file animals-14-02781-s001.zip › animals-3186857-supplementary.pdf]

Supplementary Materials: Location, Age, and Antibodies Predict Avian Influenza Virus Shedding in Ring-billed and Franklin's Gulls in Minnesota

Matthew Michalska-Smith [1,2], Eva Clements [3], Elizabeth Rasmussen [3], Marie R Culhane [3,*] and Meggan E Craft [1]

S1. (Sero)positivity by landscape variables

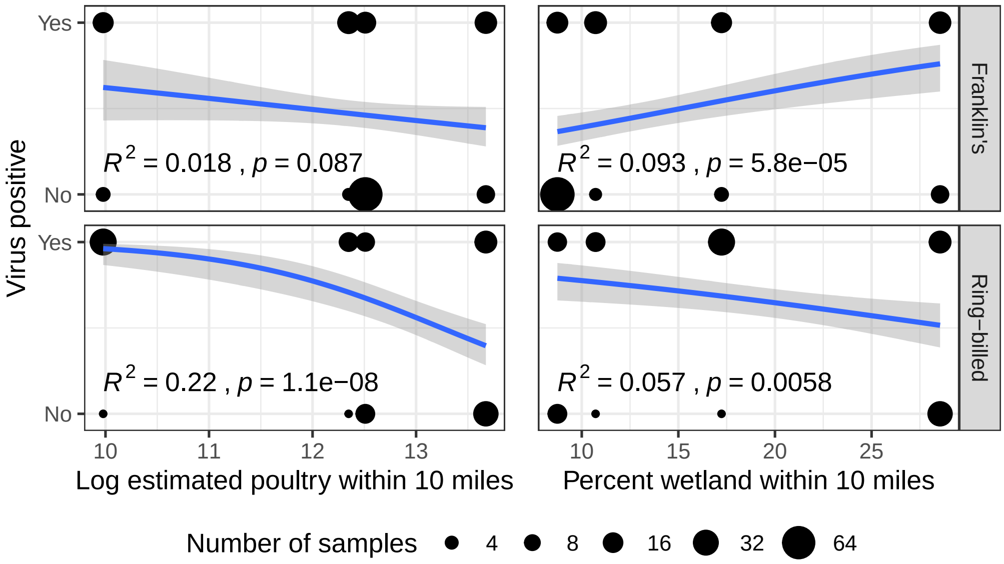

Figure S1. Binomial regressions of PCR virus detection by landscape variables, subsetting data to include only dates/sites with at least 5 birds captured from each species.

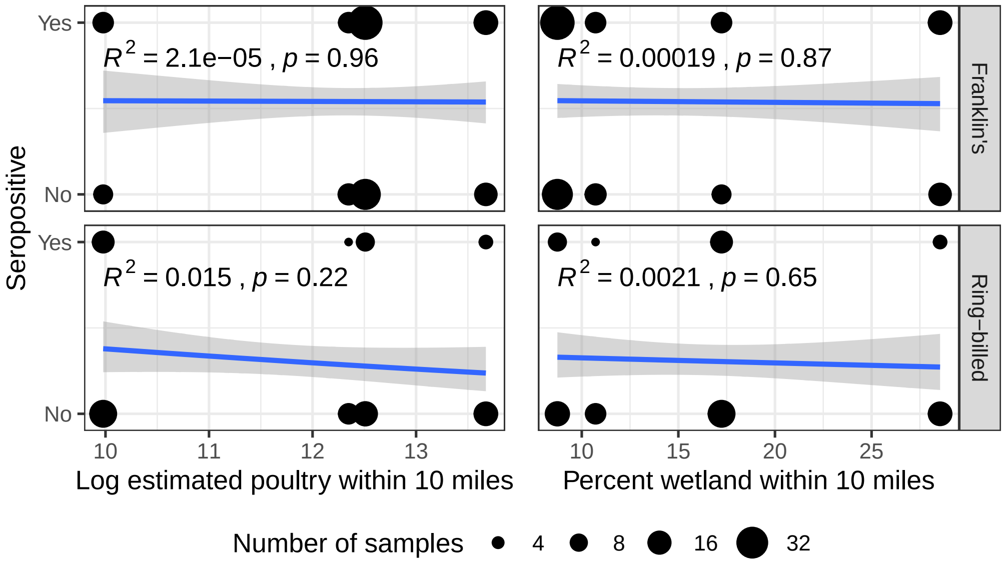

Figure S2. Binomial regressions of ELISA antibody detection by landscape variables, subsetting data to include only dates/sites with at least 5 birds captured from each species.

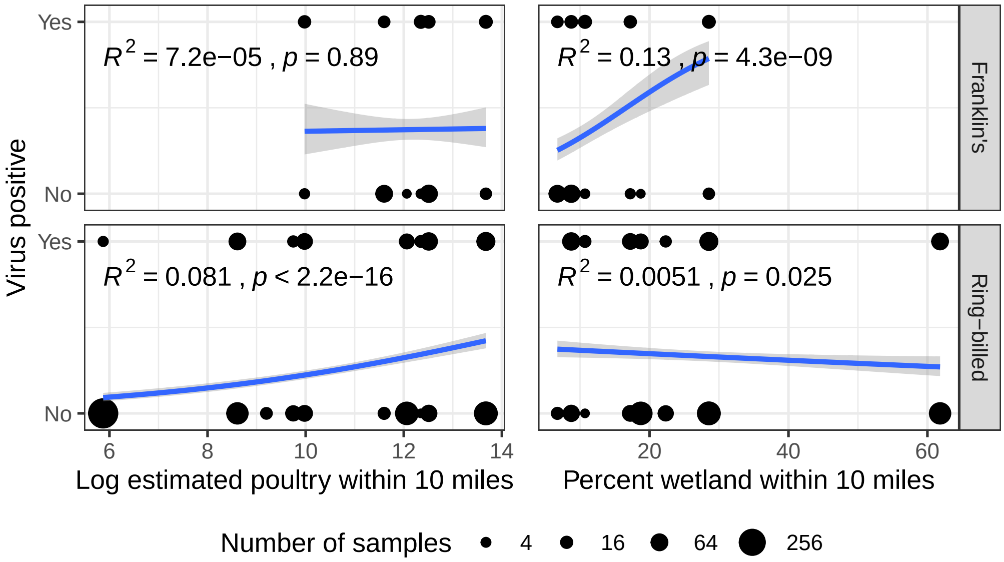

Figure S3. Binomial regressions of PCR virus detection by landscape variables, using all available samples. *N.b.* the Interstate Island Duluth location was excluded from the percent wetland regression analysis, since we were unable to standardize wetland classifications across state lines.

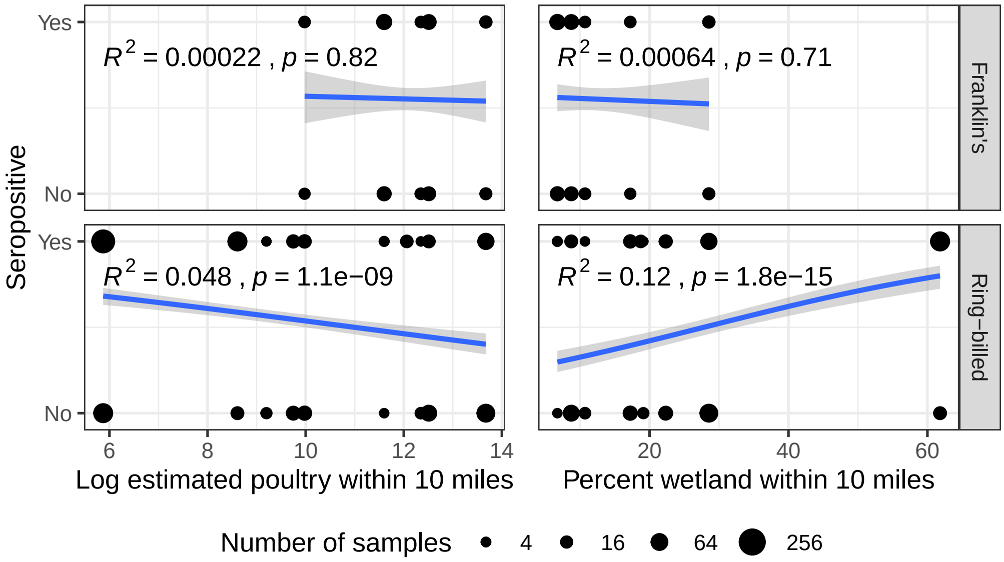

Figure S4. Binomial regressions of ELISA antibody detection by landscape variables, using all available samples. *N.b.* the Interstate Island Duluth location was excluded from the percent wetland regression analysis, since we were unable to standardize wetland classifications across state lines.

S2. Continuous response variable figures analogous to main text

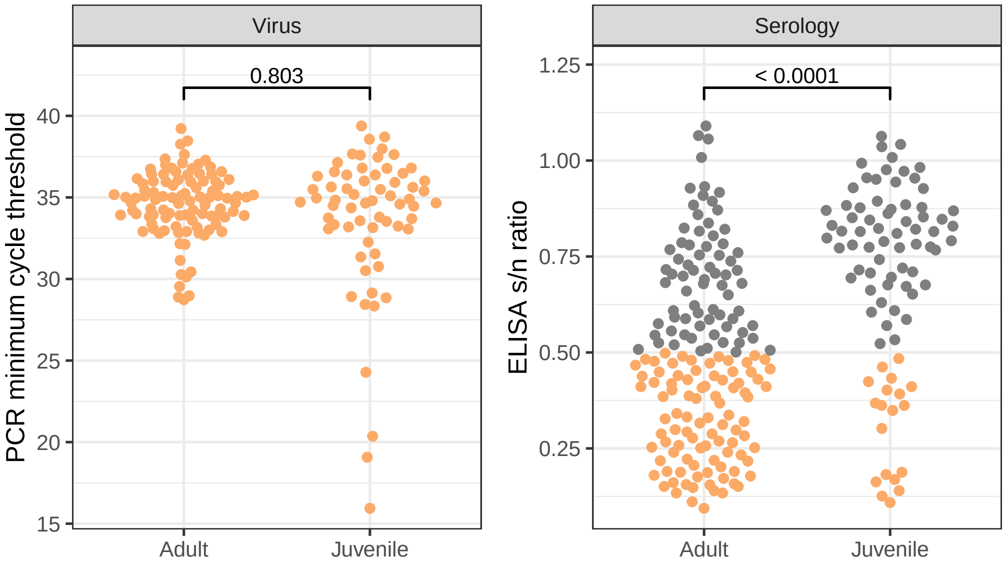

Figure S5. As Figure 4 in the main text, but using continuous response variables for each test (ELISA result-to-negative control absorbance (s/n) ratio for the serology and the minimum of the two PCR cycle thresholds (cloacal and oropharyngeal)). Reported p-values are the results of 2-sample Wilcoxon Rank Sum Tests. For the serology, both positive (yellow) and negative (gray) test results are presented, while there is no cycle threshold for negative PCR tests.

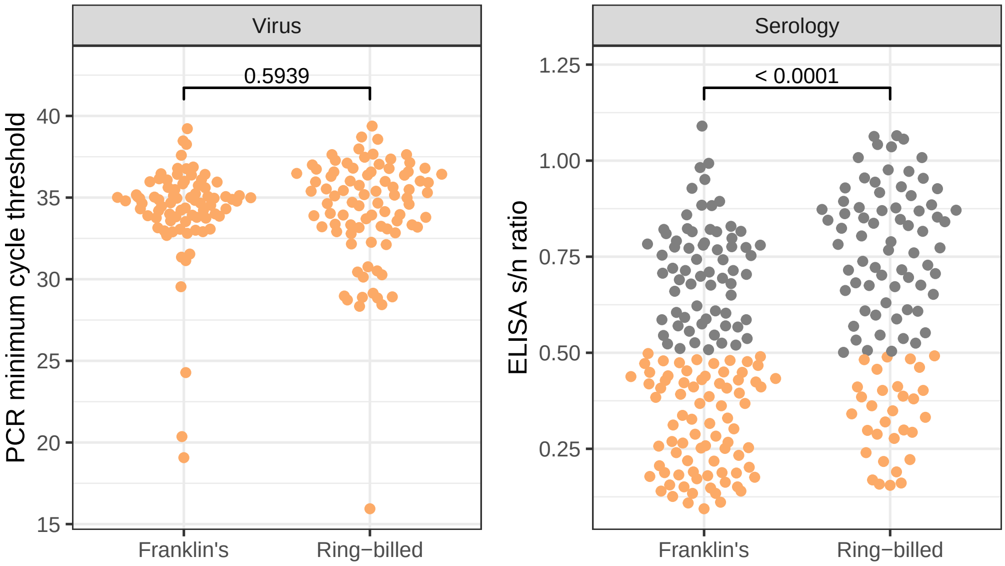

Figure S6. As Figure 5 in the main text, but using continuous response variables for each test (ELISA result-to-negative control absorbance (s/n) ratio for the serology and the minimum of the two PCR cycle thresholds (cloacal and oropharyngeal)). Reported p-values are the results of 2-sample Wilcoxon Rank Sum Tests. For the serology, both positive (yellow) and negative (gray) test results are presented, while there is no cycle threshold for negative PCR tests.

S3. Full data figures analogous to main text

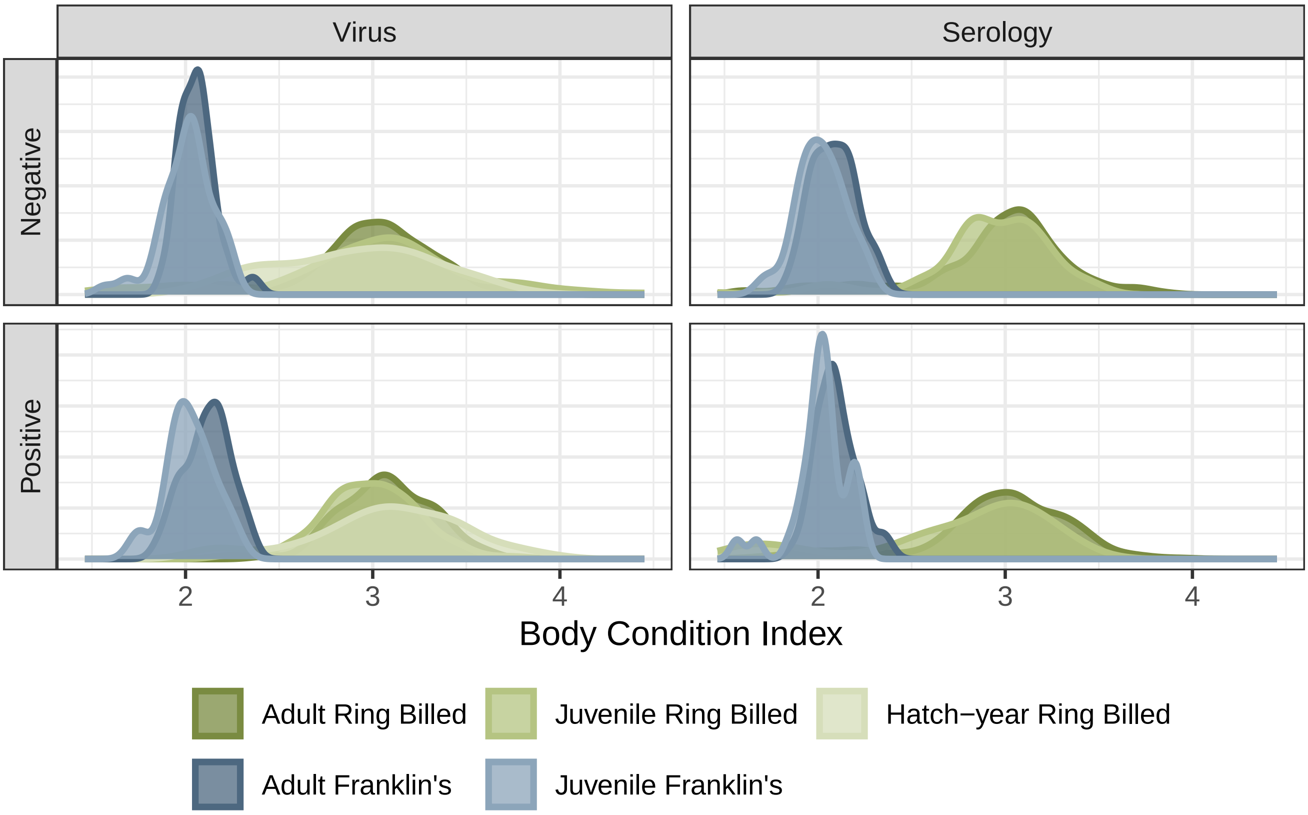

Figure S7. As Figure 3 in the main text, but using all available samples.

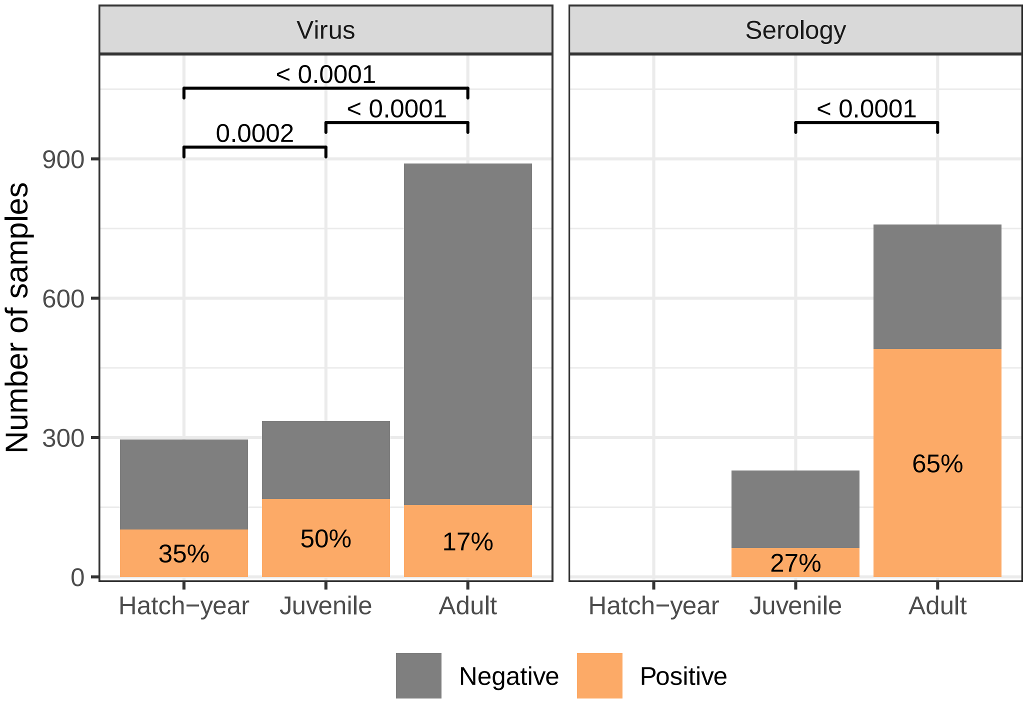

Figure S8. As Figure 2, but using all available samples. reported p-values are the results of 2-Sample Tests of Equal Proportions. N.b. serology was not conducted on hatch-year birds.

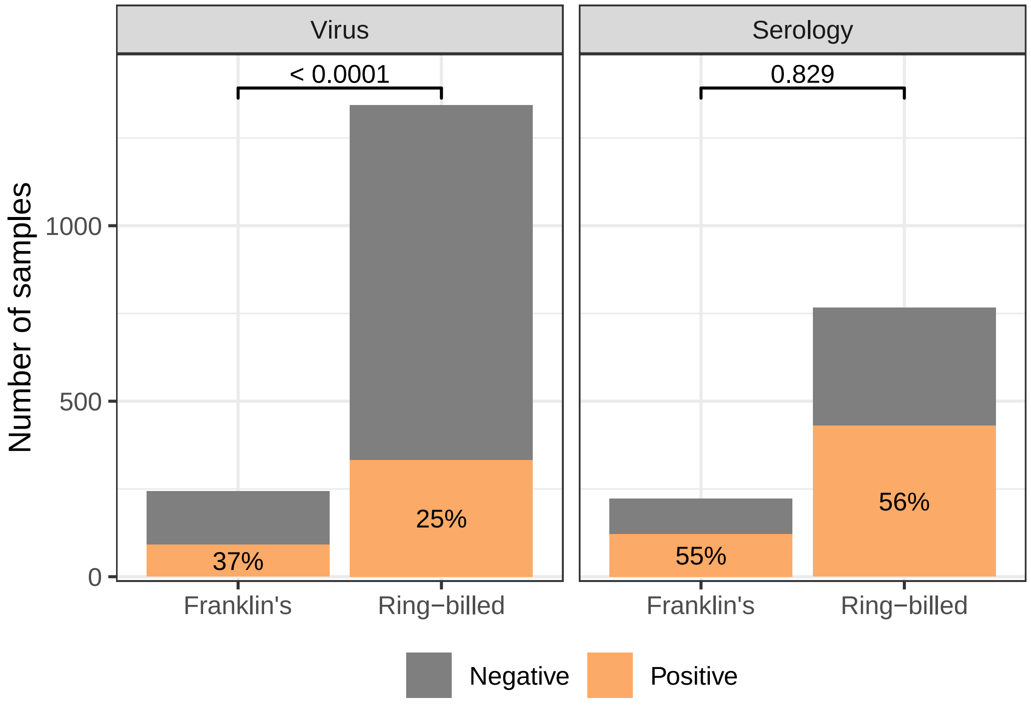

Figure S9. As Figure 3, but using all available samples. Reported p-values are the results of 2-Sample Tests of Equal Proportions.

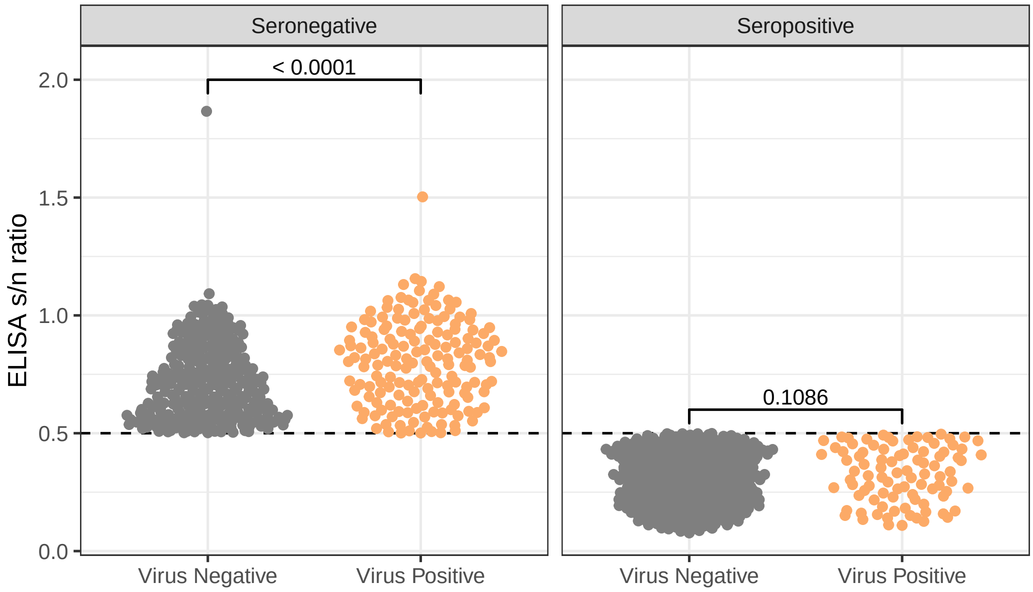

Figure S10. As Figure 4, but using all available samples. Reported p-values are the results of 2-sample Wilcoxon Rank Sum Tests.

S4. Full data figures with continuous response variables

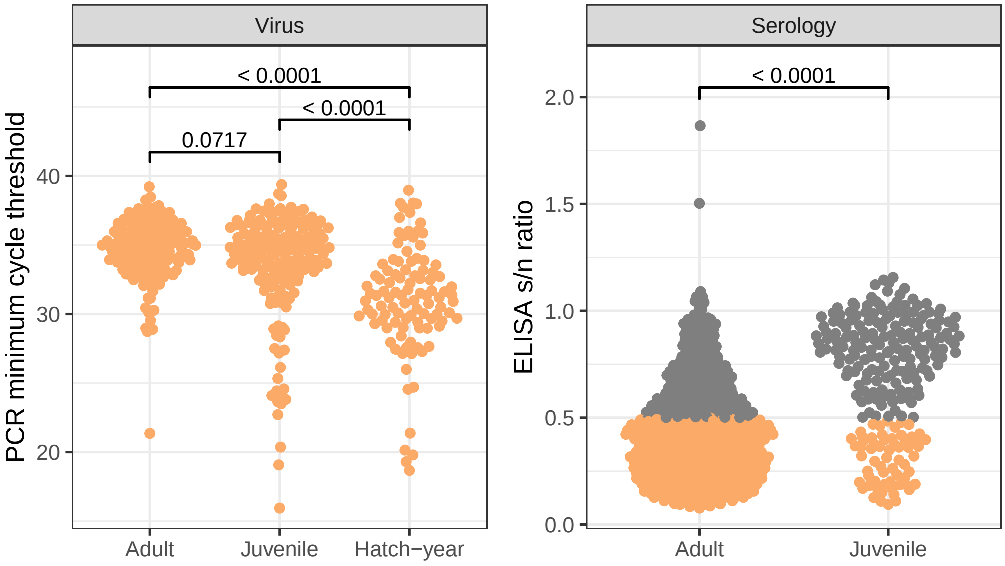

Figure S11. As Figure S8, but using all available samples. Reported p-values are the results of 2-sample Wilcoxon Rank Sum Tests.

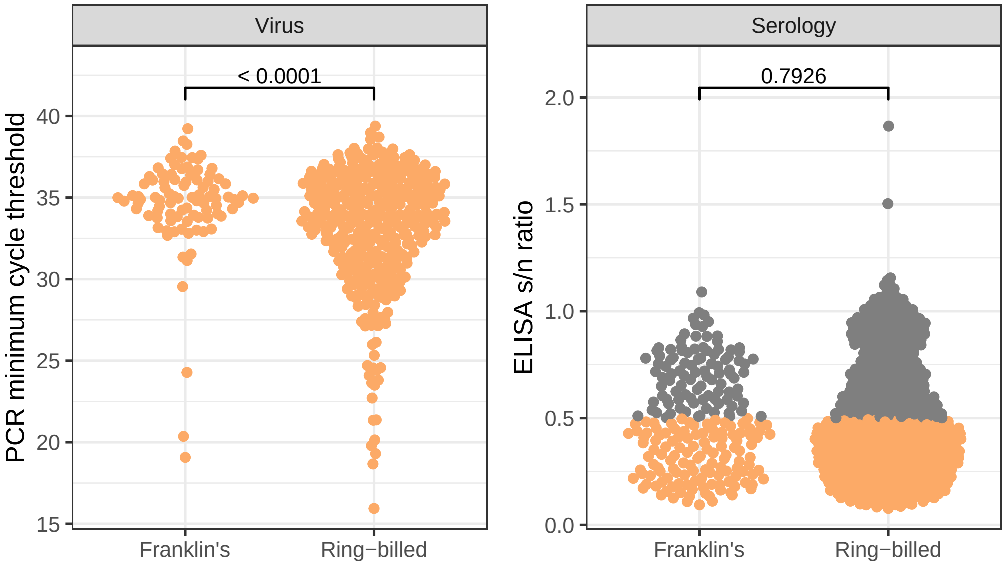

Figure S12. As Figure S9, but using all available samples. Reported p-values are the results of 2-sample Wilcoxon Rank Sum Tests.

S5. Covariate correlations

5

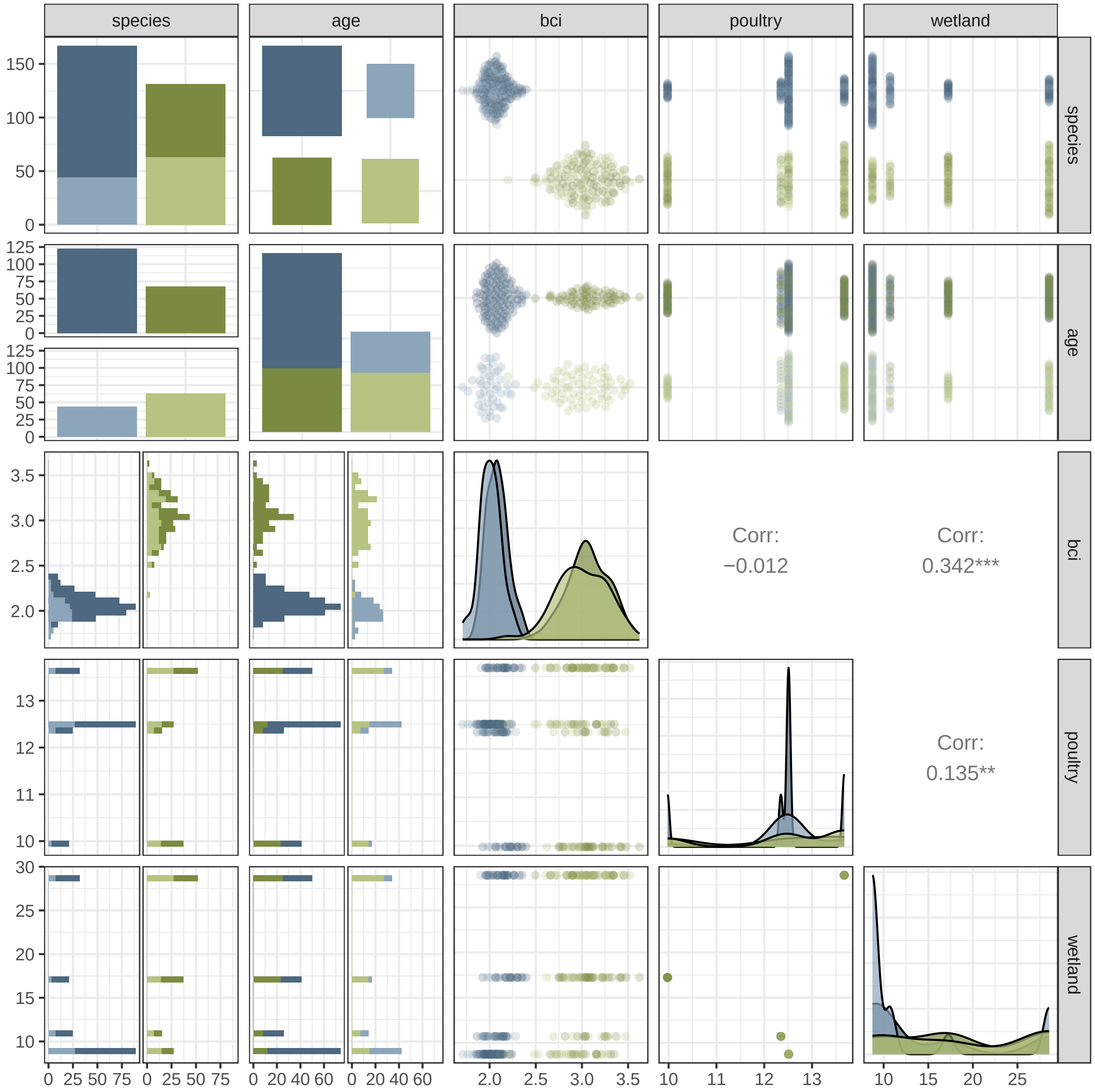

Figure S13. Correlations between all covariates used in the main text analyses, only considering the subset of samples for which at least 5 birds captured from each species on a given site/date. Covariates include gull species (Franklin's (blue) or ring-billed (green)), bird age (juvenile (lighter shades) or adult (darker shades)), body condition index (defined as the ratio of weight to the sum of head to beak length and keel length), number of poultry in production facilities within 10 miles of sampling site, and percentage of land area classified as wetlands within a 10-mile radius of the sampling site.

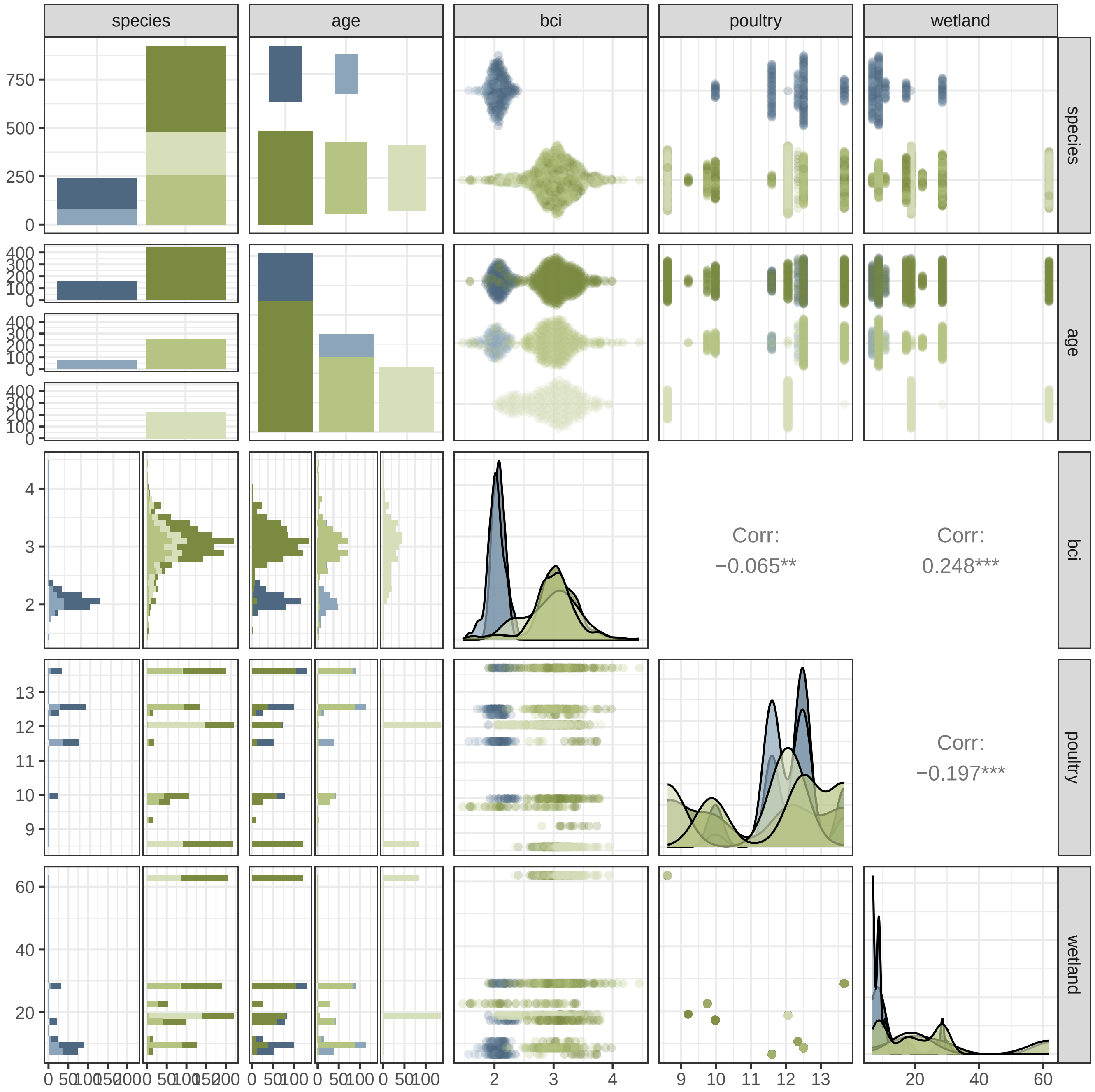

Figure S14. As Figure S16, but for all available samples. Covariates include gull species (Franklin's (blue) or ring-billed (green)), bird age (hatch-year (lightest shades; only ring-billed gulls) juvenile (intermediate shades) or adult (darker shades)), body condition index (defined as the ratio of weight to the sum of head to beak length and keel length), number of poultry in production facilities within 10 miles of sampling site, and percentage of land area classified as wetlands within a 10-mile radius of the sampling site. *N.b.* the Interstate Island Duluth location was excluded from the percent wetland regression analysis, since we were unable to standardize wetland classifications across state lines.

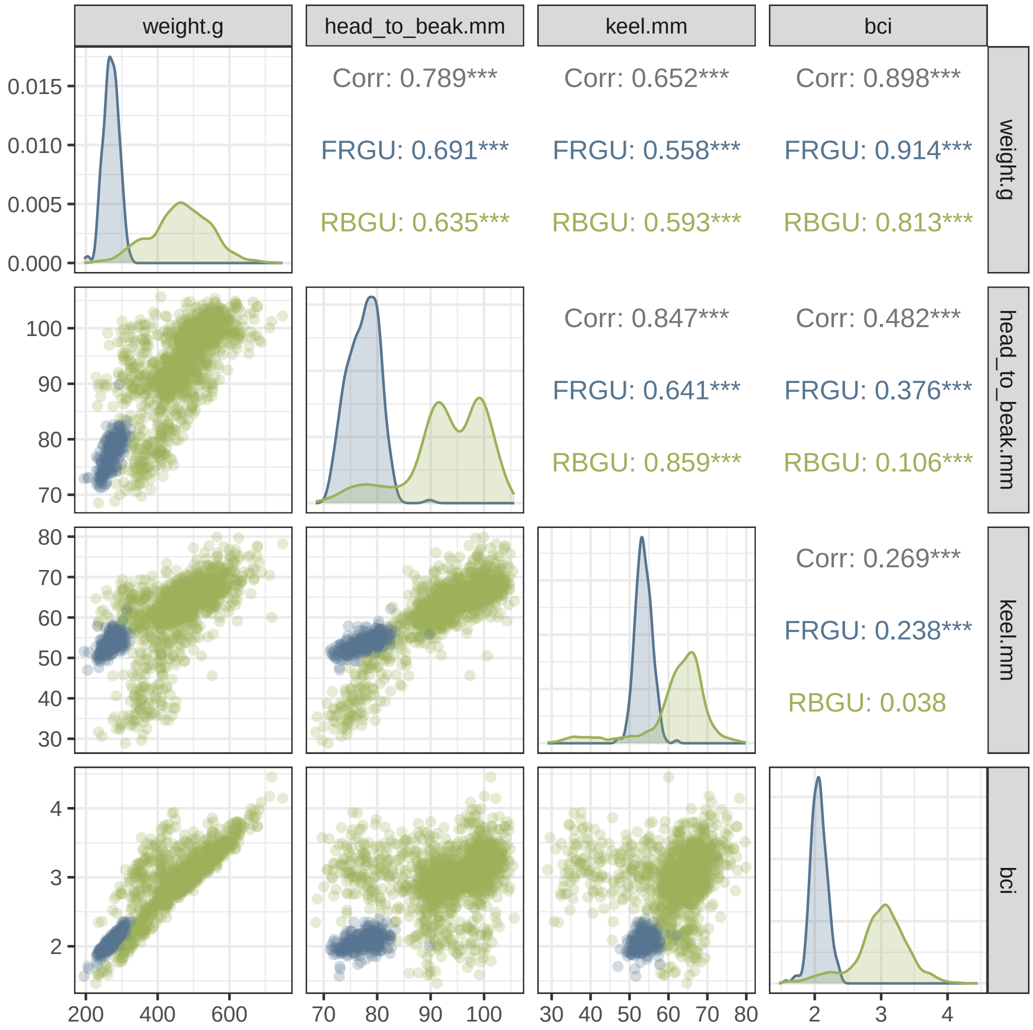

Figure S15. Correlations between morphometric measurements (weight (g), distance from back of head to beak tip (mm), and keel length (mm)) and body condition index (defined as the ratio of weight to the sum of head to beak length and keel length). Color indicates gull species (Franklin's (blue) or ring-billed (green)).
